# Restoration of motor control and proprioceptive and cutaneous sensation in humans with prior upper-limb amputation via multiple Utah Slanted Electrode Arrays (USEAs) implanted in residual peripheral arm nerves

**DOI:** 10.1186/s12984-017-0320-4

**Published:** 2017-11-25

**Authors:** Suzanne Wendelken, David M. Page, Tyler Davis, Heather A. C. Wark, David T. Kluger, Christopher Duncan, David J. Warren, Douglas T. Hutchinson, Gregory A. Clark

**Affiliations:** 1 Department of Bioengineering, University of Utah, Salt Lake City, UT 84112 USA; 2 Department of Neurosurgery, University of Utah, Salt Lake City, UT 84132 USA; 3 Department of Psychiatry, University of Utah, Salt Lake City, UT 84102 USA; 4 Division of Phys. Med. and Rehabilitation, University of Utah, Salt Lake City, UT 84132 USA; 5 Department of Orthopedics, University of Utah, Salt Lake City, UT 84108 USA

**Keywords:** Prosthetic hand, Neural interface, Motor decode, Nerve stimulation, Sensory feedback, Amputee, Neural prosthesis, Peripheral nerve, Haptics, Phantom-limb syndrome

## Abstract

**Background:**

Despite advances in sophisticated robotic hands, intuitive control of and sensory feedback from these prostheses has been limited to only 3-degrees-of-freedom (DOF) with 2 sensory percepts in closed-loop control. A Utah Slanted Electrode Array (USEA) has been used in the past to provide up to 81 sensory percepts for human amputees. Here, we report on the advanced capabilities of multiple USEAs implanted in the residual peripheral arm nerves of human amputees for restoring control of 5 DOF and sensation of up to 131 proprioceptive and cutaneous hand sensory percepts. We also demonstrate that USEA-restored sensory percepts provide a useful source of feedback during closed-loop virtual prosthetic hand control.

**Methods:**

Two 100-channel USEAs were implanted for 4–5 weeks, one each in the median and ulnar arm nerves of two human subjects with prior long-duration upper-arm amputations. Intended finger and wrist positions were decoded from neuronal firing patterns via a modified Kalman filter, allowing subjects to control many movements of a virtual prosthetic hand. Additionally, USEA microstimulation was used to evoke numerous sensory percepts spanning the phantom hand. Closed-loop control was achieved by stimulating via an electrode of the ulnar-nerve USEA while recording and decoding movement via the median-nerve USEA.

**Results:**

Subjects controlled up to 12 degrees-of-freedom during informal, ‘freeform’ online movement decode sessions, and experienced up to 131 USEA-evoked proprioceptive and cutaneous sensations spanning the phantom hand. Independent control was achieved for a 5-DOF real-time decode that included flexion/extension of the thumb, index, middle, and ring fingers, and the wrist. Proportional control was achieved for a 4-DOF real-time decode. One subject used a USEA-evoked hand sensation as feedback to complete a 1-DOF closed-loop virtual-hand movement task. There were no observed long-term functional deficits due to the USEA implants.

**Conclusions:**

Implantation of high-channel-count USEAs enables multi-degree-of-freedom control of virtual prosthetic hand movement and restoration of a rich selection of both proprioceptive and cutaneous sensory percepts spanning the hand during the short 4–5 week post-implant period. Future USEA use in longer-term implants and in closed-loop may enable restoration of many of the capabilities of an intact hand while contributing to a meaningful embodiment of the prosthesis.

**Electronic supplementary material:**

The online version of this article (10.1186/s12984-017-0320-4) contains supplementary material, which is available to authorized users.

## Background

Amputees using commercially-available mechanical or robotic prostheses do not currently receive cutaneous or proprioceptive sensory feedback from their prosthesis, nor do they have simultaneous, independent, proportional control over all the digits of the prosthetic hand and the wrist. Sensory feedback from, and dexterous control of, a prosthetic robotic hand may assist upper-limb amputees in activities of daily living (ADL), restore a sense of prosthesis embodiment, and alleviate phantom pain [1–8].

As early as 1974, amputees were instrumented with a single cuff-like electrode on their residual median nerve, which produced limited sensations in the phantom hand via electrical stimulation [3]. More recently, implanted longitudinal intrafascicular electrodes (LIFEs) were implanted into the peripheral arm nerves of several trans-radial amputees, and recordings from these electrodes provided subjects with one-degree-of-freedom (DOF) online control of a prosthesis [4]. Additionally, a limited number of sensations were evoked in the phantom hand by electrical stimulation via LIFE electrodes [4–6]. LIFE recordings were later used to achieve 3-DOF control of a prosthetic hand, including coordinated grips, and basic object discrimination was enhanced by use of two sensory percepts elicited from electrical stimulation of the peripheral nerve via LIFEs [9]. Cuff electrodes (flat interface nerve electrodes, FINEs), implanted around each of the three major residual arm nerves of an amputee, have also been used to evoke 19 sensory percepts, and these percepts have been shown to be stable for up to 2 years [10]. The Utah Electrode Array (UEA) has been previously implanted in the distal median nerve of an intact individual and used to provide 1-DOF decode and limited sensory feedback in a closed-loop interface [11, 12]. However, it is unclear if such an approach would work on transradial amputees who have modified physiology in their residual arm. Finally, a recent closed-loop system has been demonstrated in which an amputee achieved 3-DOF control of a prosthetic hand using surface electromyography (sEMG) for motor control and transverse intra-fascicular multichannel electrodes (TIMEs) implanted in residual arm nerves to provide sensory feedback in two phantom-hand locations [13].

Previously, we demonstrated that a single USEA implanted in a residual peripheral arm nerve in human amputees can be used to evoke up to 81 different cutaneous percepts on the hand and provide proportional motor control of up to two DOFs [14]. These past subjects, referred to here as S1 and S2, were each instrumented with only one USEA, implanted at the terminal end of either the residual median or ulnar nerve, respectively. Preliminary results regarding multi-USEA instrumentation in two residual arm nerves of a third subject, S3, have also been presented [15–17], demonstrating cutaneous sensory percepts spanning the phantom hand, limited 2-DOF online motor control, and basic closed-loop control.

In expansion of this work, we now present findings from two recent human subjects, S3 and S4. In addition to the use of two USEAs per subject (one in each of the median and ulnar arm nerves) for both S3 and S4, a notable improvement was achieved by implanting USEAs in S4 in the upper arm, proximal to extrinsic-hand-muscle nerve-branches. This allowed for unprecedented dexterous hand control of up to 12 DOFs (informally quantified), and generation of numerous proprioceptive sensory percepts spanning the hand in addition to many USEA-evoked cutaneous percepts, totaling up to 131 percepts overall. We also report results regarding electrode and percept stability, successful automated electrode selection prior to motor decode, and a performance comparison of different decode algorithms. Preliminary reports of some of these findings have previously appeared [15, 18–20].

## Methods

### Study volunteers

Two trans-radial amputees, referred to here as subjects S3 and S4, were recruited in 2013 and 2014 respectively, and evaluated by a physician and psychologist for their willingness and ability to participate in the study (S1 and S2, published previously [14]). S3 was a 50-year-old left-dominant male, whose left arm had been amputated several centimeters proximal to the wrist 21 years prior, following a crush injury. S4 was a 36-year-old ambidextrous male, with bilateral amputations several centimeters distal to the elbow 16 years prior, due to electrical injury. Baseline phantom limb surveys and medical histories were taken for each subject prior to the study. The surveys included assessment of the subjects’ perceived abilities to exert voluntary control over phantom movements, and perceive sensations (both painful and non-painful) on their phantom limbs. Phantom pain was assessed on the basis of the duration, frequency, and intensity of pain episodes and this assessment continued during the duration of the implant period and for several months afterward.

For the one-month period prior to the study, S3 was given a mirror box in order to practice the phantom-hand movements to be performed in the study [2]. Due to his being a bilateral amputee, S4 was unable use a mirror box and was instead given videos of hand movements to watch and imitate with his phantom hands. S3 continued his use of Gabapentin to relieve back pain throughout the study, which may affect peripheral-nerve activity. The study and consenting of human volunteers was approved by the University of Utah Institutional Review Board, the Salt Lake City Veterans Affairs Hospital Research and Development Service Center, and the Department of the Navy Human Research Protection Program.

### Device

**Fig. 1:**
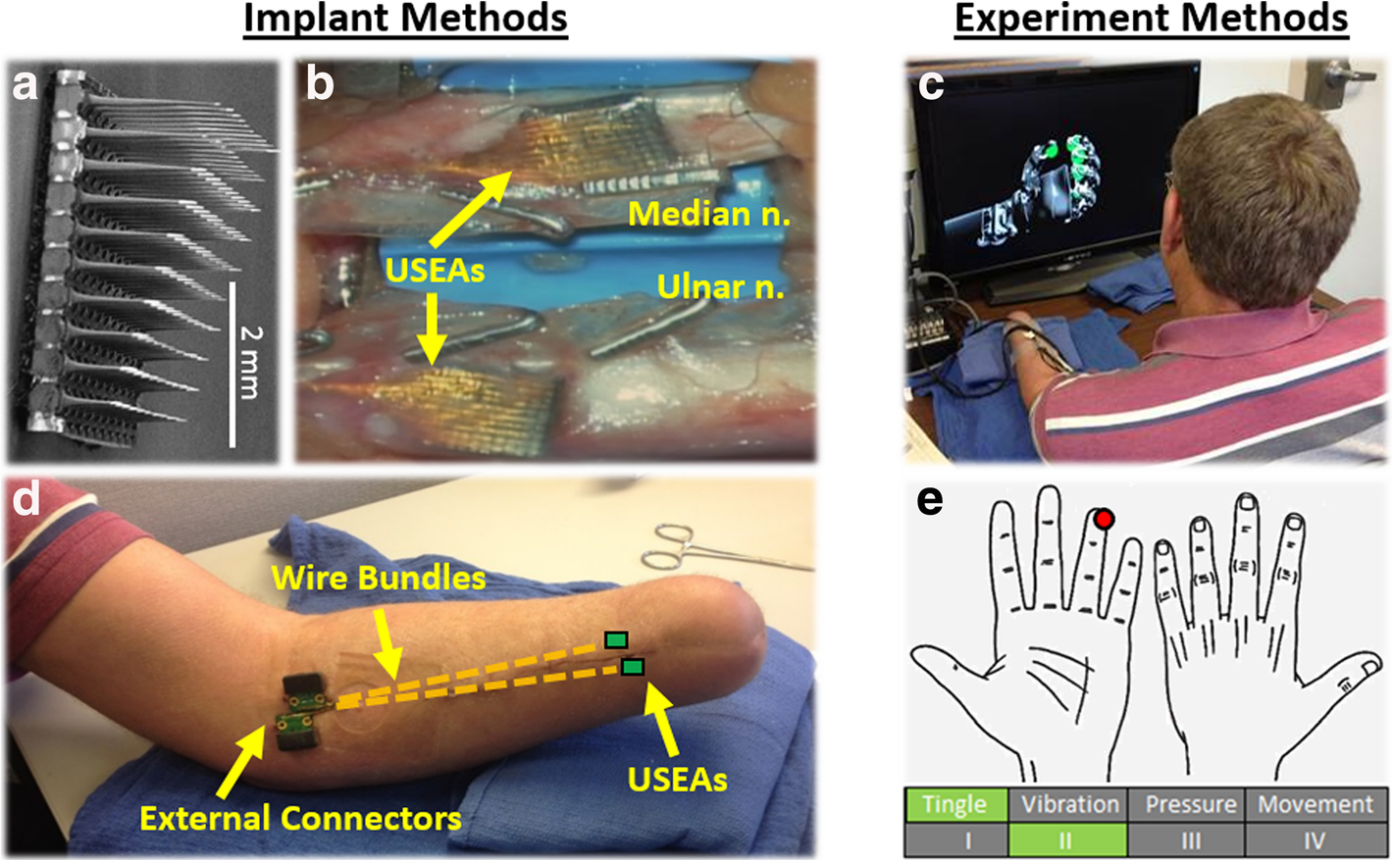
USEAs implanted in human peripheral arm nerves were used to provide amputees with multi-DOF control of virtual prosthetic hand movement and restore numerous hand sensations. **a** Scanning electron microscope image of a USEA [21]. **b** Two USEAs were implanted in each subject (S4, shown here), one in each of the median and ulnar arm nerves. An organic nerve wrap, fastened with vascular clips, enclosed each USEA. **c** USEA lead wires and ground and reference wires were connected to external connectors via a percutaneous incision (S3, shown here). **d** USEA recordings were used to provide subjects with control of movement of a virtual prosthetic hand (S3, shown here). **e** USEA stimulation was used to provide subjects with numerous sensations on the phantom hand. Subjects documented the nature of each sensation (location, quality, and intensity/size) using custom software

Two Utah Slanted Electrode Arrays (USEAs; Blackrock Microsystems, Salt Lake City, UT, USA) were implanted in each subject (one in the median nerve, one in the ulnar nerve). Each USEA consisted of 100 silicon microelectrodes arranged in a 10 × 10 grid on a 4 × 4 mm base, spaced at 400 μm, and varying in length from ~0.75–1.5 mm [21] (Fig. 1a). Of the 100 electrodes on each USEA, 96 were used to record from and/or stimulate the nerve. Four electrodes near the corners of the USEA were used as an on-array electrical reference [22], and two separate looped platinum wires served as off-array electrical reference and ground leads. All implanted electrodes were wired via a percutaneous incision to a custom-developed printed circuit board designed to allow attachment to data acquisition and stimulation hardware via a ZIF-Clip-96 connector cable (Tucker-Davis Technologies Inc., Alachua, FL, USA).

### Surgical procedures

Prior to, and for several days following the implant procedure, subjects were given a prophylactic antibiotic (100 mg minocycline, 7 days b.i.d., starting the day before the implant surgery) which potentially improves the quality of chronic neuronal recordings [23]. Under general anesthesia, two USEAs were surgically implanted into each subject—one in the residual median nerve and one in the residual ulnar nerve (Fig. 1b). In S3, both USEAs were placed in the lower arm, approximately 2 cm proximal to the amputation neuroma (Fig. 1c). This distal location was used in S3 as an initial precautionary measure, because nerves were not functionally attached at the distal implant locations. Hence, any nerve resection there would not compromise essential motor or sensory function. In S4, both USEAs were placed in the upper arm, approximately 2 cm proximal to the medial epicondyle. Importantly, the USEAs in S4 were proximal to many motor and sensory nerve-branch points, including branches to extrinsic hand muscles, thereby potentially providing a greater richness in motor and proprioceptive nerve fiber access.

For S3, the surgical procedure involved the passage of the unprotected USEAs through a trocar from the percutaneous site to the implant site, which resulted in damage to four of the electrodes on the median nerve implant (and no documented damage to the ulnar nerve implant). A different USEA passage method was devised for S4, which involved securing the arrays inside a plastic tapered carrier for protection before passing them under the skin. There is no indication that any electrodes were damaged using this revised USEA passage method in S4.

In both subjects, the epineurium was dissected from the surface of the nerves prior to pneumatic insertion of the USEAs [24]. The USEA wire bundle, ground, and reference wires were sutured to the epineurium (8-0 or 9-0 nylon suture), and a protective collagen wrap (AxoGen Inc., Alachua, FL, USA) was placed around the nerve, USEAs, and reference/ground wires. The wrap was secured with vascular clips and sutured to the epineurium for stability. After tourniquet removal, subjects were administered 0.1 mg/kg of dexamethasone intravenously to potentially mitigate the foreign body response and improve neural recording capability [25, 26].

Percutaneous wire-passage sites were re-dressed as needed throughout the study, on at least a weekly basis. Antibiotic wound dressings (Biopatch, Ethicon US LLC, Somerville, NJ, USA) were placed directly over the percutaneous site throughout the study duration to reduce the risk of infection, although S4 did experience an infection from which he fully recovered (potentially due to an implant-related hematoma and/or via the percutaneous wire-passage site).

After several weeks (4 weeks for S3, 5 weeks for S4), the USEAs were surgically explanted. In S3, the USEAs and neuromas were removed with the arrays still intact for histological analysis [27]. In S4, only the USEAs were removed due to their placement midway along the nerves in the upper arm.

### Experiment setup

Subjects returned for the first experimental session within 4 days of the USEA implant surgery. Experimental sessions were 1–6 h in duration, and were performed 3–5 days per week for 4 weeks for S3 and 5 weeks for S4. Experimental sessions typically included testing impedances of all USEA channels at the beginning of each session, followed by a recording/decoding session, a stimulation session, or both.

### Impedance testing

The impedance of each electrode on each USEA was measured in saline prior to implantation via one-week soak-testing using a custom-built impedance tester, at 1 kHz [28]. Impedances were also measured shortly before pre-implant sterilization using the NeuroPort System (Blackrock Microsystems, Salt Lake City, UT, USA) at 1 kHz. Impedance testing was subsequently performed in vivo at the beginning of each experimental session using the NeuroPort System at 1 kHz.

Impedance measurements were used to identify failed USEA electrodes/channels as well as to monitor the over-time stability of working electrodes. We defined failed channels as those which had an impedance greater than or equal to 500 kΩ. Non-failed channels were defined as channels that never had an impedance value above 500 kΩ across the implant duration. For each implanted USEA, we tested the null hypothesis that the number of failed USEA electrodes in a session does not change significantly across the implant duration, using a two-tailed Spearman’s rank correlation. Additionally, for each implanted USEA, we tested the null hypothesis that the impedance value for non-failed electrodes does not change over time using a Friedman test followed by a post-hoc two-tailed Wilcoxon’s signed-rank test between the first and last post-implant impedance testing sessions. The Friedman test served as a screening criteria; the Wilcoxon’s signed-rank test was performed only if the Friedman test was statistically significant. Given the use of a cut-off value, and that impedance values vary on a non-negative, logarithmic scale, we utilized nonparametric tests that did not assume normality.

### Recording/decode

Neural data collection was performed using the 128-channel NeuroPort System for S3 and either the NeuroPort System or the 512-channel Grapevine System (Ripple LLC, Salt Lake City, UT, USA) for S4. Continuous neural signals were band-pass filtered with cutoff frequencies of 0.3 Hz (1st-order high-pass Butterworth filter) and 7500 Hz (3rd-order low-pass Butterworth filter), and digitally sampled at 30 kHz. A digital high-pass filter was applied to sampled recordings (250 Hz, 4th-order Butterworth filter), and multi-unit activity was extracted by detecting threshold crossings of an adaptive, automated threshold, set to approximately negative 6 times the root mean square (RMS) of the signal. Spike-event times from each electrode were binned into 33.3-millisecond windows and converted into firing rates, which were then used as inputs to train and test a modified Kalman filter decode algorithm. In this application of the Kalman filter, we modified the decoder to impose a limit of −1 to 1 in order to prevent the decoder from exceeding the limits of the robotic or virtual hand (that is normalized from −1 to 1). Outputs of trained decode algorithms were used to provide the subjects with real-time control of the position of a simulated hand in a virtual environment [29] (Fig. 1d).

Here we define a degree-of-freedom (DOF) as the motion in a digit or the wrist in a single linear or rotational axis in either direction. Thus, a single DOF includes deviation from a rest position in both flexion and extension direction (e.g., a positive value indicates motion in the flexion direction and a negative value indicates motion in the extension direction). We define an individual ‘movement’ as a DOF including the directional component for each DOF (e.g., flexion and extension); consequently, there were twice as many possible individual ‘movements’ as possible individual DOFs. Although the virtual hand used in experiments had 24 actuating joints, interphalangeal joints were tied to the metacarpal phalangeal joints, giving the virtual hand a total of 12 DOF (flexion/extension of digits 1–5; adduction/abduction of digits 1, 2, 4, and 5; flexion/extension, ulnar/radial deviation, and pronation/supination of the wrist). The virtual model did not include adduction/abduction of digit 3.

To train the decode algorithm, the subjects were instructed to imitate with their phantom hands a series of single-DOF virtual-hand movements shown on a computer screen while USEA recordings were collected and saved. Training sets included 5 to 10 trials of each movement, with each movement trial lasting for 1 to 2 s (complete training session generally lasting 5–10 min). The time from training-set completion to online decode testing was typically no longer than 5 to 10 min. Training was conducted at the start of a given day’s experimental recording session.

During individual training motions, the experimenters manually selected a subset of electrode channels and movements by viewing electrode maps of spiking activity and selecting the electrode channels with greatest apparent correlation and specificity to a single movement. These electrodes were then used as inputs for training online decodes, whereas electrode channels with little or no firing that was correlated preferentially with single movements were excluded. On-line automated channel selection had not yet been implemented at the time of these experiments, but has been implemented since [18, 19].

Data from online training sessions were analyzed offline in order to compare performance of electrode selection methods, decode algorithms, and movement constraints. Electrode selection methods included manual and automated selection. Manual selection was performed during online decode experiments, and the same manually-selected electrodes were later used for offline decodes. Automated electrode selection was performed only for offline decodes, and involved selecting the electrodes that produced a correlation of at least 0.5 between firing rate and training movement cue position.

For both online and offline decodes, at least five training trials, along with the associated firing rates of selected channels, were used to establish the coefficients of the decode algorithm. For offline decodes, the remaining five trials were used for validation testing of the algorithm. Offline decodes were performed using both automated electrode selection and manual electrode selection for both a standard Kalman filter algorithm [30], or the ReFIT Kalman filter algorithm [31] (available for S4 only). These algorithms were chosen due to the stability of output in the presence of a noisy input signal. For each of up to 12 DOFs trained on, correlation coefficients between the decoded position and the intended position (half-cycle of a sine wave) were computed across all five trials. For each decode algorithm/configuration, the mean of the correlation coefficients across individual DOFs was computed to allow for comparison for S4. In the automated channel selection method, the algorithm selected only those electrodes that produced a correlation of at least 0.5 between firing rate and training movement cue position. Further details of the decoding algorithm are discussed elsewhere [19, 30].

Formal assessment of online decode performance was carried out via a virtual target-touching task. Specifically, one or more spherical virtual target(s) was positioned away from the resting position of one or more digit(s)/wrist along the arc of movement. The subject was then instructed to move the specified digit(s)/wrist inside the radius the spherical target(s) for at least 250 ms while keeping the other DOFs in resting position. The virtual targets did not exclude the virtual fingers, and fingers could pass all the way through the spheres. Typically, a target diameter 15% of total range of motion was chosen during formal assessments. A trial was considered failed if the subject did not complete the task within a 30-s time-limit. In the case of closed-loop trials, a misclassification of the target’s distance by the subject was also considered a failure. After successful completion of a trial, virtual targets were automatically reset to their resting positions, and the subject was required to maintain all degrees-of-freedom in their resting positions for 1 s before another trial was presented. To verify proportional control while using a unchanging set of decoding parameters, the subjects performed a similar task with targets located and held at several different positions along the trajectory of each DOF.

Informal, “freeform” sessions in which intent was not objectively specified and hence errors were not directly measurable were also performed. RMS values of the decode output for all DOFs were calculated during a rest period. If the value of the decode output exceeded +/− 6*RMS during periods of intentional movement for a particular DOF, it was considered controlled by the subject.

### Stimulation

Electrical stimulation was performed using the IZ2–128 System (Tucker-Davis Technologies Inc., Alachua, FL, USA) for S3 and either the IZ2–128 System or the Grapevine System (Ripple LLC, Salt Lake City, UT, USA) for S4. For all USEA stimulation, biphasic, cathodic-first pulses were used (typically 200 μs width for each phase, 100 μs inter-phase interval). When a percept was evoked by USEA stimulation, subjects indicated the perceived location, quality, and intensity or size of the percept on an image of a hand using custom software (Fig. 1e). Subjects were instructed to select the percept quality from a list of descriptors (e.g. ‘tingle’, ‘vibration’, ‘pressure’, ‘movement’, ‘hot’, ‘cold’) or to create and use their own descriptors as necessary.

Full-USEA stimulation threshold maps were collected on weeks 1, 2, 3, and 4 for S3, and on weeks 2 and 5 for S4. For these maps, the threshold current (in μA) required to evoke a sensation via stimulation of each electrode was determined. Thresholds were defined as the minimum current level at which a subject repeatedly perceived stimulation-evoked percepts. For these mappings, biphasic, 200-μs stimulus pulses (with a 100-μs inter-phase interval) were delivered via single electrodes at 200 Hz for a 200-ms-duration train (the 200 Hz frequency was chosen empirically on the basis of the subjects’ ability to quickly reach threshold). The stimulation trains were initiated either by the experimenter or self-initiated by the subject via clicking a mouse button.

Full-USEA threshold mapping sessions began by sequentially stimulating each electrode on the USEA individually with a low-amplitude stimulus (e.g., 2 μA), while documenting electrodes for which either a percept was evoked, or for which the voltage between the stimulating electrode and return electrode (looped platinum ground wire) did not return above the safety level of −0.6 V before the end of the interphase interval [32]. These electrodes were excluded from subsequent stimulation, whereas each of the remaining electrodes on the USEA was again sequentially stimulated at an incrementally higher current level. This pattern was repeated at increasing current levels until either there were no remaining un-mapped electrodes, or the current reached a maximum threshold amplitude (varied between 35 μA and 120 μA depending on the subject and the session), at which point all remaining electrodes were excluded.

For both subjects, full-USEA threshold mapping routines were performed at multiple times during the study, allowing for temporal stability analysis of the nature of percepts evoked by each electrode. Specifically, we quantified each USEA’s percept stability based on the percentage of electrodes on that USEA for which the evoked percept changed either location or quality between two consecutive full-USEA threshold-mapping sessions. For this analysis, a change in percept location was defined as a transition between any of 12 hand location categories (front/back of palm, and front/back of each of the 5 digits). A change in percept quality was defined as a transition between selected percept quality descriptors. For S3, we computed the across-week mean of the number of electrodes that had a change in either percept quality or location from week to week. For S4, full-USEA threshold maps were collected only on week 2 and week 5 due to time restrictions, and the percentage of electrodes which had a change in either location or quality between these two sessions was quantified.

Additionally, we tested the null hypothesis that stimulation threshold currents for each electrode do not change significantly over time, using either a Friedman test with a post-hoc two-tailed Wilcoxon’s signed-rank test between the first and final threshold mapping sessions (for S3), or a two-tailed Wilcoxon’s signed-rank test (for S4, because there were only two full-USEA threshold mapping sessions). For S3, the Friedman test served as a screening criteria such that the Wilcoxon’s signed rank test was only performed if the Friedman test revealed a statistically significant temporal trend in the perceptual threshold. For each full-USEA threshold mapping session, we also calculated the percentage of median- and ulnar-nerve evoked percepts that were within the expected nerve-location distribution (based on muscular and cutaneous innervations documented in intact hands and arms [33, 34]).

### Closed-loop control

For S3, stimulation was delivered via a single electrode on the ulnar-nerve USEA during an online, one-DOF decode of simultaneous four-finger flexion produced via recordings on the median-nerve USEA. In a target acquisition task similar to others used for online decode testing, USEA-evoked sensory feedback was delivered whenever the virtual fingers were within virtual spherical targets, producing a basic sense of virtual-object touch. Virtual targets were presented in a pseudorandom order in two different locations: ‘close’ or ‘far’, representing finger contact positions that were either close to, or far from, finger resting positions (equivalent to grasping a large-diameter or small-diameter object, respectively). For a successful trial, the subject was required to move the virtual fingers into the boundary of the virtual target and stay within the target zone for 250 ms and then correctly indicate whether the target was ‘close’ or ‘far’. Failed trials were those in which the subject either indicated the wrong distance to target, or failed to maintain 250 ms of consecutive contact with the virtual target before the 30-s time-limit. Importantly, these trials were performed in the absence of visual feedback from the computer monitor, presumably limiting feedback regarding contact with the virtual object to that evoked by USEA stimulation. The subject was given verbal feedback as to the correctness of his answer by the experimenters after each trial.

## Results

### Electrode impedances

**Fig. 2:**
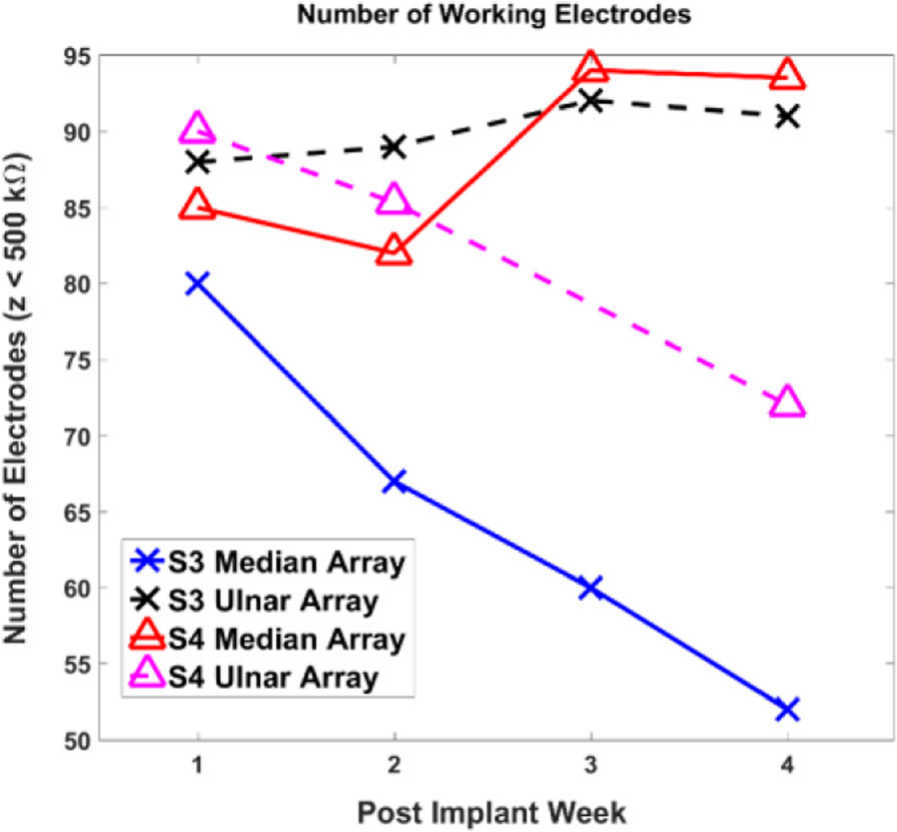
Number of working electrodes (impedance <500 kΩ) of 96 total recording electrodes per array over time for S3 and S4. The number of working electrodes for the ulnar array of S3 and median array of S4 is relatively stable, whereas the number of working electrodes steadily declines for the median array of S3 and ulnar array of S4

Implanted USEA electrodes had mixed resistance to failure over time. One array in each subject maintained a steady and high number (> 80) of working electrodes (impedances <500 kΩ). The other array in both subjects showed a steady decline over the implant duration (Fig. 2). The point of failure (e.g. electrode metallization, electrode insulation, array wiring, connector pin, etc.) for a given electrode was not determined.

For each of the four USEAs, impedances on non-failed channels (i.e., with impedance never ≥500 kΩ) changed significantly over time (*p < 0.0001,* Friedman test, 2 dof (S4), 3 dof(S3); see Table 1). Post-hoc testing between the first and final post-implant sessions revealed a significant pairwise drop in impedance for electrodes on S4’s median-nerve USEA (*p < 0.0001;* two-tailed Wilcoxon’s signed-rank test), but did not reveal a statistically significant pairwise change for the remaining 3 USEAs.

**Table 1:** Weekly medians and interquartile ranges (IQR) of electrode impedances for all USEAs. Post-hoc testing between the first and final post-implant sessions revealed a significant pairwise drop in impedance for electrodes on S4’s median-nerve USEA (*p < 0.0001;* two-tailed Wilcoxon’s signed-rank test, denoted with ^a^), but did not reveal a statistically significant pairwise change for the remaining 3 USEAs (*p = 0.82* S3 ulnar, *p = 0.12* S3 median, *p = 0.99* S4 ulnar)

USEA	Number of non-failed electrodes	Week 1	Week 2	Week 3	Week 4
S3 Median Array	41	96 kΩ (65 kΩ)	81 kΩ (41 kΩ)	89 kΩ (36 kΩ)	99 kΩ (47 kΩ)
S3 Ulnar Array	81	171 kΩ (79 kΩ)	141 kΩ (107 kΩ)	110 kΩ (52 kΩ)	188 kΩ (96 kΩ)
S4 Median Array	60	167 kΩ (117 kΩ)	186 kΩ (70 kΩ)	337 kΩ (71 kΩ)	85 kΩ (69 kΩ) ^a^
S4 Ulnar Array	59	127 kΩ (69 kΩ)	143 kΩ (99 kΩ)	194 kΩ (101 kΩ)	118 kΩ (96 kΩ)

### Decoding USEA recordings allowed intuitive control of many movements

USEA recordings from individual days provided subjects with online proportional control of multiple DOF of the virtual hand. In formal evaluations, S4 controlled up to 5 DOFs independently, including flexion/extension of the thumb, index, middle, and ring fingers, as well as the wrist. Both subjects reported the experience of moving their fingers to be emotionally meaningful. In an informally-assessed online decode setting, S4 was able to control up to 12 DOFs, including flexion/extension of all 5 digits; abduction/adduction of the thumb, ring, and little fingers; and wrist flexion/extension and rotation (Additional file 1: Video 1). Additional file 1: Video 1.Supplemental video showing a 12-DOF online decode “freeform” session for S4. In this video, S4 was controls 12 DOFs in a “freeform” session. S4 was instructed to move the hand in any way he desired. Movements available to the subject included flexion/extension of all 5 digits; abduction/adduction of the thumb, ring, and little fingers; and wrist flexion/extension and rotation. (MP4 13,805 kb)


**Fig. 3:**
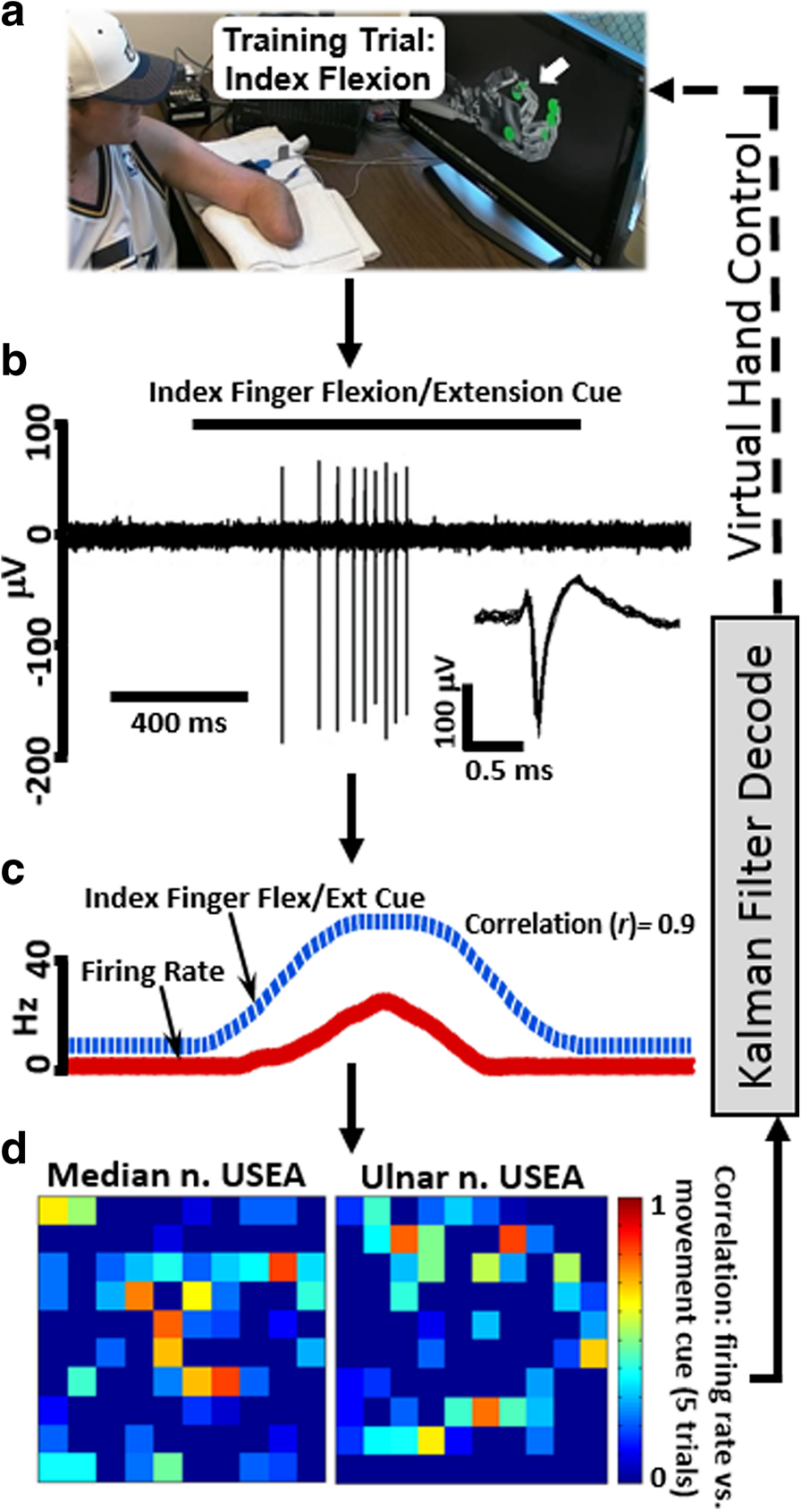
USEA recordings were collected during a training session and used to train a decode (either a standard Kalman filter or a ReFIT Kalman filter). Subjects were then given online control of the virtual hand via real-time output from the trained decode. **a** Training data was collected by recording via USEAs while the subject imitated pre-programmed, single-DOF or multi-DOF virtual hand movements with their phantom hand. **b** Neuronal spiking was observed during intended movements (inset shows neuronal action potential waveforms). **c** For each trial, the firing rate on a given electrode was computed and compared to the movement cue position via correlation. **d** The correlation between firing-rate and movement cue was determined for each USEA electrode across many trials of a given movement, and electrodes with high correlations and independent activation for specific movements were selected as input for an online decode

**Fig. 4:**
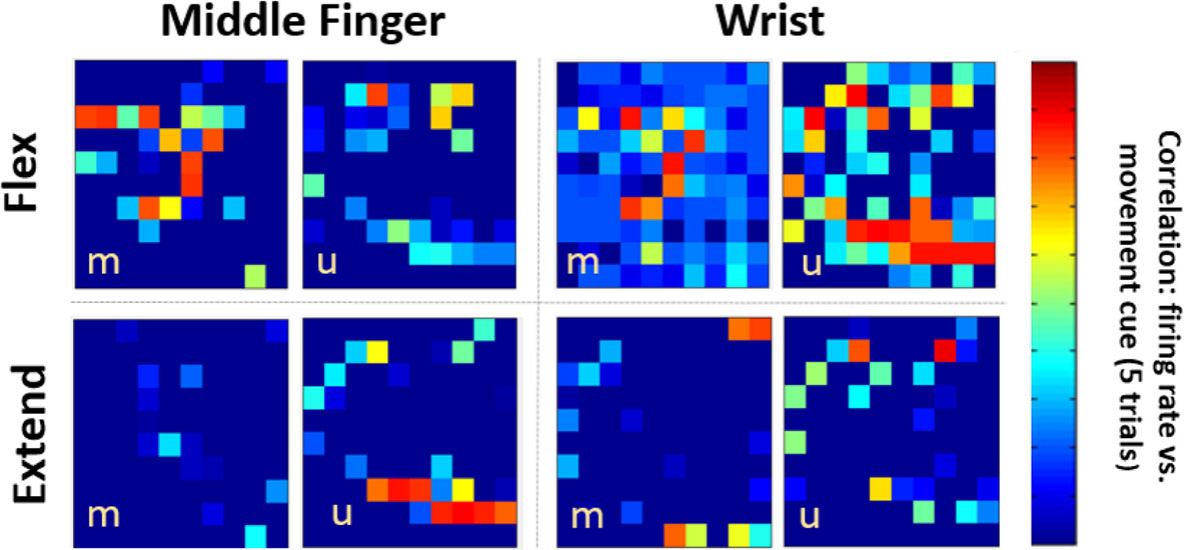
Distinct patterns of USEA electrodes with firing rates correlated to movement position (across two electrode arrays, median array (m) and ulnar array (u)) are apparent for different movements (i.e. unique sub-populations of axons fire with specific movement efforts). Shown here are the patterns of the firing rates during movement cues for 2 DOFs (middle finger and wrist pitch) for S4. To see this figure in color, go online

In S4, multiple USEA electrodes displayed neuronal spiking activity concurrent with movement cues (Fig. 3). The spike morphology and timing suggests that the activity was of neuronal rather than muscular origin (Fig. 3b, inset). Additionally, the pattern of movement-correlated firing on USEA electrodes for a given individual training movement was unique, and differed for different individual movements (Fig. 4). For S3, while neuronal and EMG spiking recorded from nearby muscles contributed to real-time decodes, EMG signals were dominant input recorded from the USEAs, typically limiting the performance of S3 decodes to 1–2 DOF (data not shown).

**Fig. 5:**
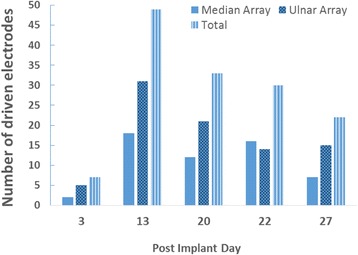
Number of electrodes with driven units for S4. Electrodes with activity correlated to volitional movement (*r* ≥ 0.5) were tabulated periodically throughout the experiment duration. The total number of driven electrodes peaks on post implant day 13, then steadily decreases over time

USEA neural recordings demonstrated poor stability over 4 weeks for S4. The number of electrodes with activity correlated to movement (“driven electrodes”) peaked on day 13, and decreased until little neural activity was detected on day 30 (Fig. 5). Despite this, USEA recordings on individual days provided sufficient source of information for decoding.

**Fig. 6:**
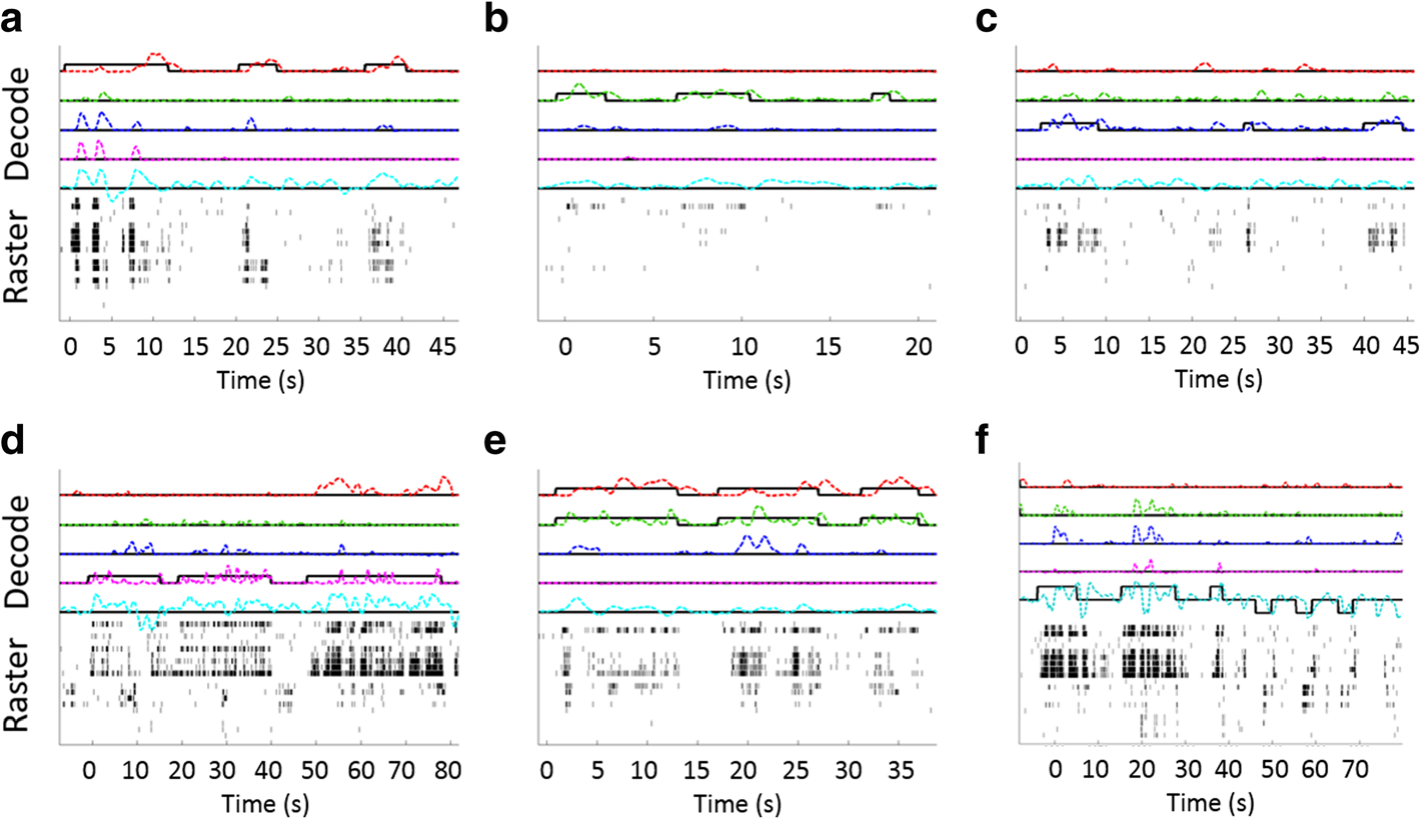
Decode output and raster plot during a 5-DOF target touching task for S4. Each figure shows the target position (solid black line) and decode output (dashed and colored line) on the top 5 lines. DOFs displayed in order from the top are thumb flex, index flex, middle flex, ring flex, and wrist flex/extend. Displayed below in black hash marks is the raster plot from 19 electrodes shown (15 from the median nerve array and 4 from the ulnar array). **a** thumb flexion targets **b** index finger flexion targets, **c** middle finger flexion targets, **d** ring finger targets, **e** thumb-index pinch target. Note that the subject was able to perform this combination movement despite training only single single-DOF training movements. **f** wrist flexion and extension targets

Formal assessments of online decode performance were carried out via a target-touching task. S4 demonstrated independent control of up to 5-DOFs, including flexion of the thumb, index, middle, and ring fingers, as well as the wrist (20/21 successful trials, median trial time 6.76 s, IQR 5.23 s). See Fig. 6 for the raw 5-DOF decode output and spike raster during several trials of each DOF tested. Notably, S4 was able to perform novel combination movements (e.g., thumb-index pinch) during multi-DOF online Kalman-filter decodes that had been trained on only single-DOF training movements (Fig. 6 inlay e). Thus, dexterous, multi-DOF control can be achieved using a limited set of simplistic training data.

**Fig. 7:**
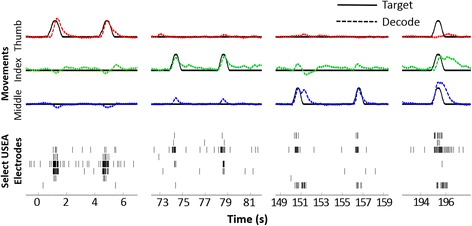
S4 tracked the position of three different moving virtual targets with the thumb, index, and middle fingers of a virtual prosthetic hand and then tracked the combined movement of all three targets with at least the middle finger and index finger. The top three traces depict the target location (solid line) and the subject-controlled, decoded virtual finger location (dashed line) for the thumb, index, and middle fingers. The subject independently tracked single thumb, index, and middle finger targets (depicted from left to right, respectively), and simultaneously tracked at least the middle and index finger targets in combination even though training data included no combination movements. The lower portion of the figure shows a raster indicating the times of recorded spike events from 8 selected median or ulnar nerve USEA electrodes during this task. The firing rates of spike events on these electrodes are uniquely tuned to different movements. To see this figure in color, go online

Proportional position control was formally verified for a 4-DOF online decode in S4 by presenting targets at two different distances (‘near and ‘far’) on different trials for each DOF (40/40 successful trials, median trial time 4.025 s, IQR 4.035 s). See Additional file 2: Video 2 for an example of 4 DOF proportional control target-touching task in S4. For S3, proportional control was verified for a 2-DOF decode (46/52 successful trials, median trial time 4.03 s, IQR 11.89 s). S4 also performed a target-tracking task in which he was instructed to follow virtual targets with specific DOFs as the targets moved in virtual space. Specifically, during a 3-DOF decode, S4 tracked targets independently with the thumb, index and middle finger, followed by combined-DOF target tracking (Fig. 7).Additional file 2: Video 2.Supplemental video showing an example of the target-touching task. To verify simultaneous and individual control of multiple DOF, a target-touching task was designed. In this task, one or more spherical virtual target(s) was positioned away from the resting position of one or more digit(s)/wrist along the arc of movement. To demonstrate proportional control, targets were placed in one of two different positions: “Near” targets were positioned at 25% flexion and “far” targets were positioned at 75% flexion. A successful trial resulted when the subject moved the specified digit(s) inside the radius the spherical target(s) for at least 250 ms while keeping the other DOFs in resting position. The virtual targets did not exclude the virtual fingers, and fingers could pass all the way through the spheres. To provide visual feedback, the target spheres change color from red to green when the desired finger enters the target sphere. The target radii were set to be 15% of the arc of motion in one direction. A trial was considered failed if the subject did not complete the task within a 30-s time-limit. After successful completion of a trial, virtual targets were automatically reset to the resting positions, and the subject was required to maintain all degrees-of-freedom in their resting positions for 1 s before the next trial was presented. This video shows an example of a target-touching task in which proportional position control was formally verified for a 4-DOF online decode in S4. Several examples of target-touching trials for 4 DOFs, including the thumb, index, middle, and ring fingers, at “near” and/or “far” distances are shown. (MP4 3493 kb)

Informal sessions where the subject was allowed to control all 12 available DOF were also performed (Additional file 1: Video 1). Because the subject controlled the hand under “freeform” conditions in which intent was not objectively specified and hence errors were not directly measureable, this is considered an informal result. However, due to the cross talk between several degrees of freedom, it is unlikely that the subject could have completed a formal 12-DOF target touching task.

### Offline decode performance

With this approach, the automated selection algorithm selected 49 USEA electrodes for an offline decode of a training set from S4, in contrast to the 24 electrodes previously selected with manual electrode selection for this training set.

The highest offline performance for a 12-DOF decode resulted from using automated electrode selection, a standard Kalman filter, and limiting movement to flexion-ranges only (i.e., digits were not allowed to extend in relation to baseline position). Specifically, the mean correlation between the intended position and decoded position across all 12 DOFs improved from 0.28 to 0.53 when automated electrode selection and flexion-only constraints were used (see Table 2). The correlation coefficient between the decoded finger position and the intended finger position generally decreased as the number of DOFs of the offline decode increased.

**Table 2:** The best 12-DoF offline decode performance resulted from automatic electrode selection and constraining the training and testing movements to be in the flexion range only. Mean correlation coefficients between predicted position and actual position were computed across each DOF of a 12-DOF offline decode.

		Electrode selection	
Constraints		Manual (24)	Automatic (49)
	All movements	0.28	0.38
	Flex range only	0.5	0.53

Flex-range-only constraints involved limiting digit movement to be forward from baseline position only, whereas flex-and-extend allowed movement both forward and backward from the baseline position. Manual electrode selection (24 electrodes selected) was performed by experimenters during online collection of the data, whereas automated electrode selection was performed offline using a thresholding algorithm

**Fig. 8:**
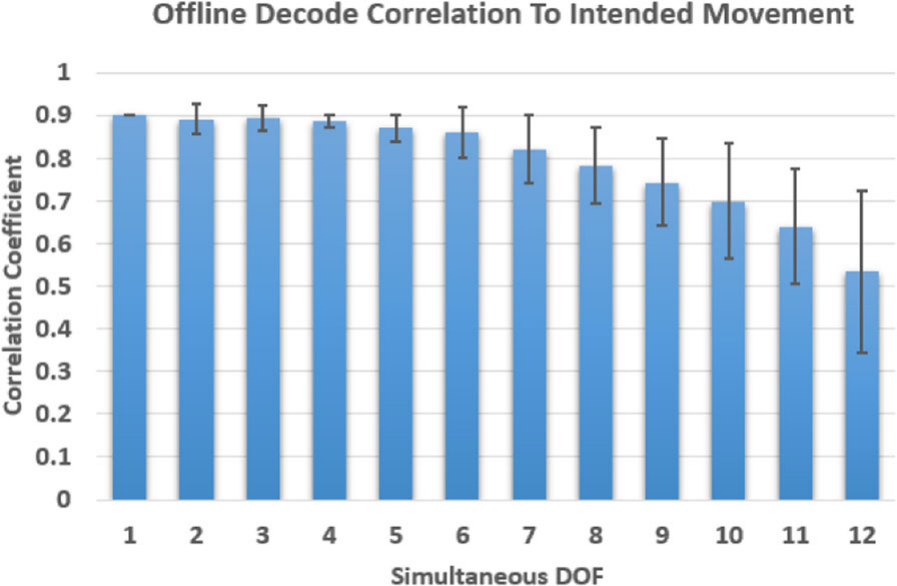
“Best case scenario” for multi-DOF offline decode. Bar chart depicts the correlation coefficients between predicted and attempted training movements of the most correlated movements in a multi-DOF offline decode of a single data set. The data set contained 8 trials of each DOF where 4 trials were used for training the decoder and 4 trials were used for testing. The solid bar represents the mean correlation coefficient of the highest correlated N movements, where N is the number of simultaneous DOF tested (whiskers are standard deviations)

Offline analysis was also performed on all possible DOFs in order to determine the “best possible” multi-DOF decode (Fig. 8). Correlation coefficients between the decoded movement and intended movement were calculated for the top N simultaneous DOFs tested. Mean correlation coefficient values were greater than 0.8 for up to 7 simultaneous DOFs indicating a high level of independent DOFs could potentially be achieved during an online decode.

### USEA microstimulation produced numerous sensations spanning the hand

For each subject, microstimulation via USEA electrodes produced nearly 100 or more unique proprioceptive and cutaneous percepts that spanned the phantom hand, providing a rich selection of percepts potentially useful as feedback from a prosthetic limb. Importantly, subjects described many of the evoked sensations in a positive manner and sometimes asked for repeated delivery of pleasurable stimuli.

**Fig. 9:**
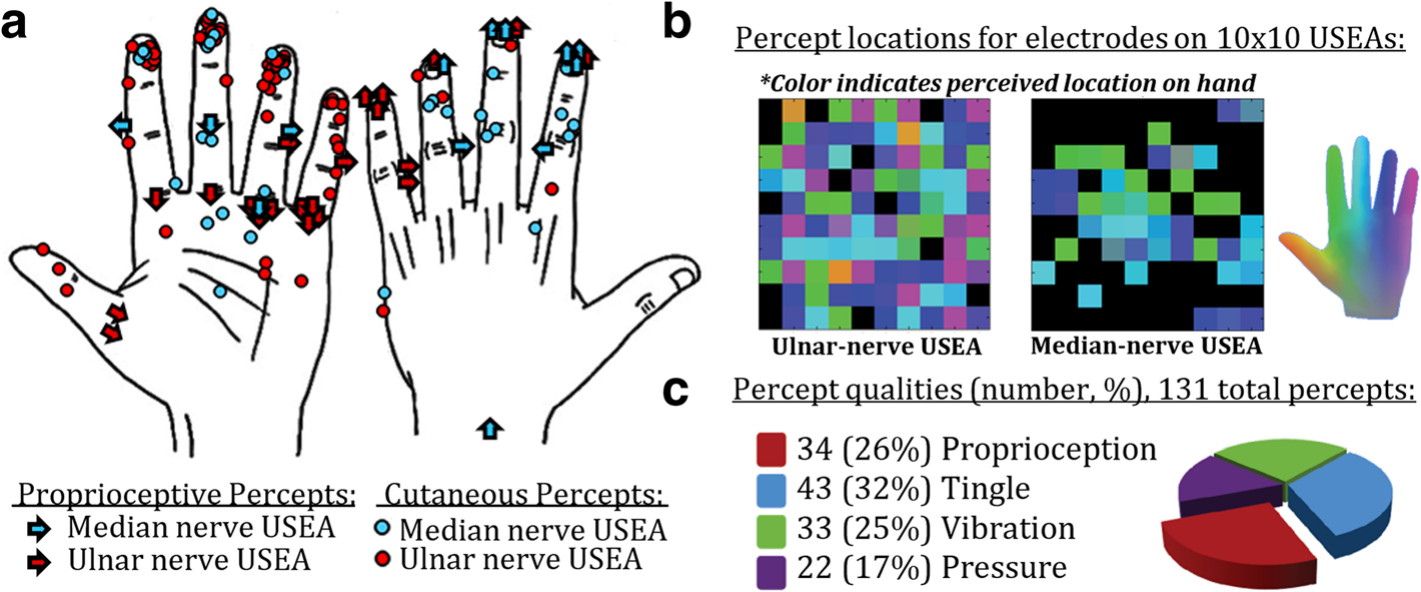
USEA microstimulation provided a rich selection of percepts of various qualities and locations spanning the phantom hand (S4 shown here). **a** Stimulation of individual electrodes via two USEAs restored 131 percepts across the phantom hand, including both proprioceptive and cutaneous percepts (collected over a 2-day period). Numerous cutaneous percepts were evoked on each digit and the palm, and proprioceptive percepts were restored for 17 different movements, including flexion and extension of each finger and flexion of the thumb. For proprioceptive percepts, upward arrows indicate extension, whereas downward arrows indicate flexion. **b** 131 electrodes across the 10 × 10 USEAs evoked the percepts shown in part A, with no apparent somatotopic arrangement across the nerve cross-section. **c** Evoked percepts were of various qualities, with 26% of evoked percepts described as proprioceptive, and 74% of evoked percepts being cutaneous (including ‘tingle’, ‘vibration’, and ‘pressure’). To see this figure in color, go online

In S4, 131 of 192 (68%) USEA electrodes produced proprioceptive or cutaneous sensory percepts spanning the hand (Fig. 9a), and in S3, 97 of 192 (51%) USEA electrodes produced sensory percepts (primarily cutaneous). Percepts were evoked using different electrodes across the slanted 10 × 10 USEA. There was no apparent somatotopic arrangement across the nerve cross-section; however, we often observed fascicular organization (Fig. 9b). Subjects also successfully discriminated among sensory percepts of different locations and qualities (a preliminary report for S3 has been provided [15], and a comprehensive report across multiple subjects is pending future publication).

Proprioceptive percepts for S4 included 17 unique perceived phantom hand movements (i.e., proprioceptive percepts), including flexion and extension of each finger; adduction and abduction of the index, ring, and little fingers; thumb flexion; and wrist extension. In S3, a proprioceptive percept was evoked only once (presumably due to implant location).

Cutaneous percepts were of many qualities, including ‘pressure’, ‘vibration’, ‘tingle’, and ‘sting’ (Fig. 9c; ‘sting’ was described only by S3). Many percepts were naturalistic and enjoyable to the subjects (e.g., ‘vibration’ and ‘pressure’), whereas some percepts were undesirable or non-naturalistic (e.g., ‘sting’, and ‘tingle’).

**Fig. 10:**
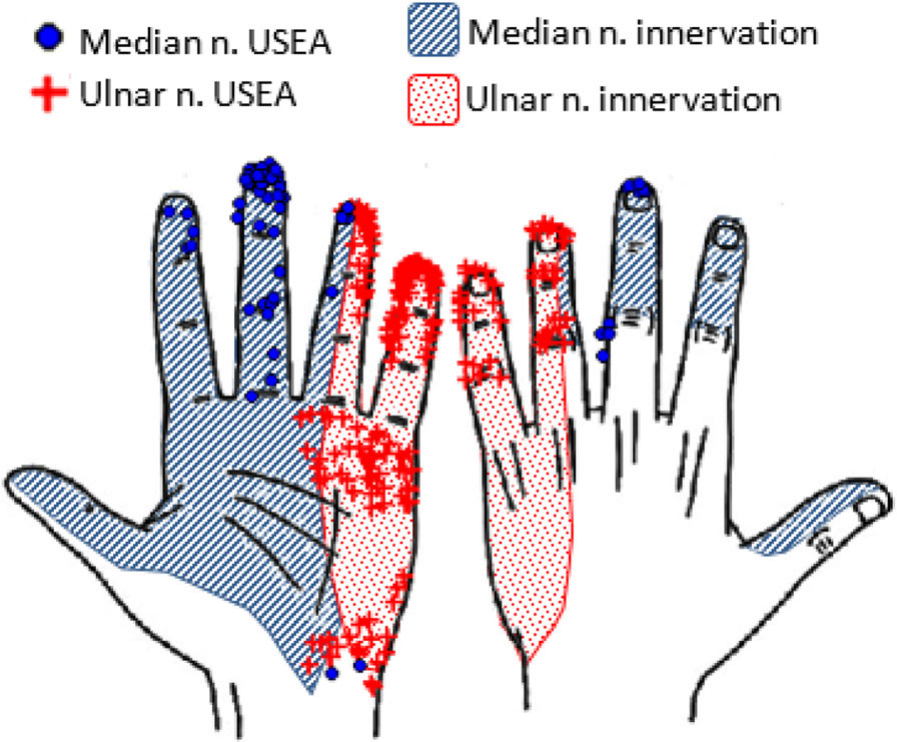
Percepts evoked by median and ulnar nerve USEAs are generally within the established intact-hand innervation regions for each nerve. For the example shown (S3, week 2), 92% and 89% of median-nerve-USEA- and ulnar–nerve-USEA-evoked percepts are within their expected distributions, respectively. To see this figure in color, go online

We compared subjects’ perceived percept location distributions for median- and ulnar-nerve percepts with the anatomically-determined median and ulnar innervation distributions of an intact hand reported in literature. For S3, on weeks 1–4, respectively, a total of 84, 90, 86, and 95% of median- and ulnar-USEA percepts were within the expected anatomical innervation regions of the hand (Fig. 10). For S4, on week 2 and week 5, respectively, 63% and 75% of median- and ulnar-USEA percepts were within their expected innervation regions (including the unique innervations for proprioceptive vs. cutaneous percepts).

For both subjects, the location and quality of percepts evoked by single electrodes was generally stable during 3–4 h experimental sessions. However, single-electrode percepts often changed location and/or quality across weeks. Specifically, for S3, across-week means of 91 and 78% of ulnar- and median-USEA electrodes evoked percepts that changed either location or quality in a one-week period, respectively (percentages are based on the 43 ulnar- and 17 median-nerve USEA electrodes that evoked percepts on all 4 weeks). For S4, 83% of the 12 median-nerve USEA electrodes that evoked percepts both on week 2 and week 5 changed either location or quality across this three-week period. Importantly, no percepts were evoked via ulnar-nerve USEA stimulation on week 5, possibly due to infection-related swelling or USEA movement.

**Fig. 11:**
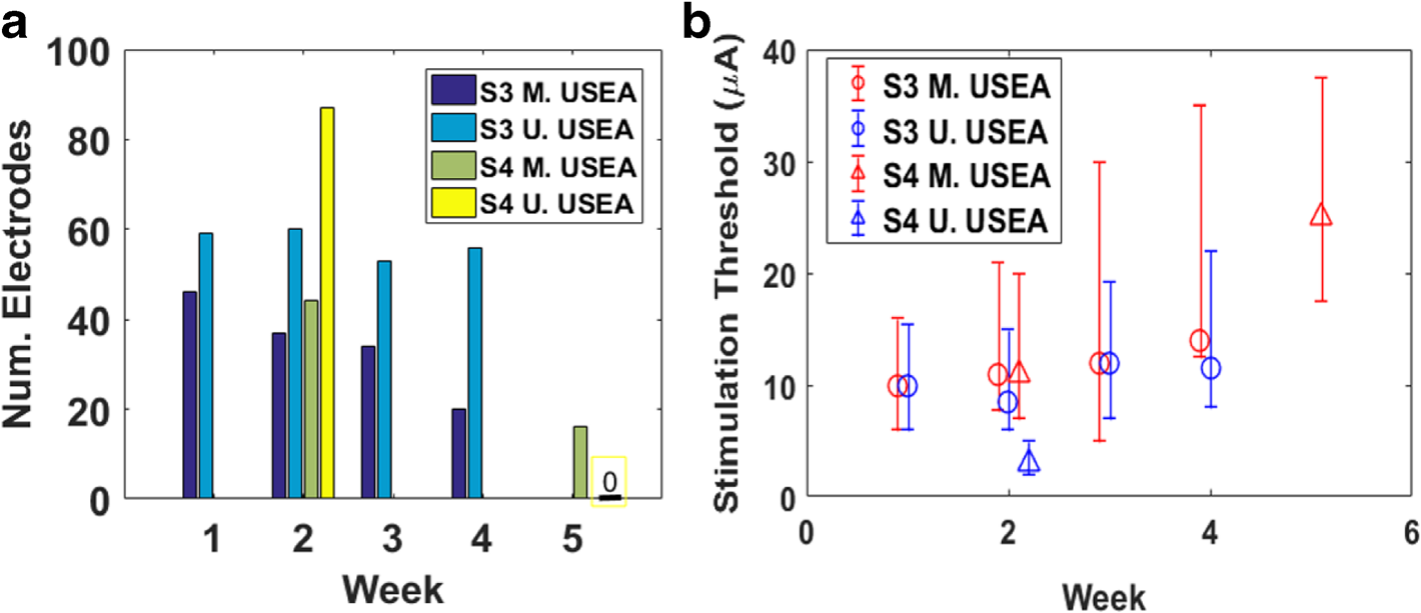
**a** The number of electrodes which evoked a sensory percept for each USEA across the implant duration (max amplitude, 120 μA, pulse duration of 200 μs). Note that perceptual thresholds for S4 were not tested on weeks 1, 3, and 4. Also, on week 5, S4’s ulnar n. USEA did not evoke any sensory percepts. **b** Weekly median and interquartile range boundaries threshold amplitudes across electrodes on each USEA. Outliers not shown

Median stimulation thresholds (and interquartile ranges) for each USEA across the implant duration are provided for both subjects in Fig. 11. For the 43 ulnar-nerve USEA electrodes on S3 that evoked percepts on all 4 weeks, threshold amplitudes changed significantly over time (*p < 0.01*, Friedman test). A post-hoc contrast test showed that stimulation thresholds tended to increase on these electrodes between week 1 and week 4 (*p < 0.01*, two-tailed Wilcoxon’s signed-rank test). Similar significant increases were evident for the 17 median-nerve USEA electrodes that evoked percepts on all 4 weeks for S3 (*p < 0.01*, Friedman test, and *p < 0.01*, post-hoc two-tailed Wilcoxon’s signed-rank test).

For S4, stimulation thresholds for full USEAs were mapped only on week 2 and week 5, due to limitations on experiment time. Notably, none of the electrodes on the ulnar-nerve USEA evoked percepts on week 5. For the 12 median-nerve USEA electrodes that evoked percepts on both week 2 and week 5 there was not significant evidence of changing thresholds over time (*p* = 0.11, two-tailed Wilcoxon’s signed-rank test).

### USEA-evoked sensations can be used for closed-loop control

During two closed-loop target-touching sessions, S3 used a cutaneous sensation on his ring fingertip (evoked by stimulation of a single ulnar-nerve USEA electrode) as feedback to determine the location of the target in virtual space which was placed in either close to the finger tips from the neutral start position (‘close’), or further along the arc of flexion of the finger tips (‘far’). In this task, S3 controlled flexion/extension of fingers 1–4 (fingers linked together into 1 DOF, decode via median-nerve USEA recording, driven by both neural and EMG signals). In the absence of visual feedback from the computer monitor, the subject successfully moved the fingers into the target region and identified the location (‘close’ or ‘far’) of virtual targets in 41/47 trials. See Table 3 for a confusion matrix of session 2. In order to successfully perform this task, S4 used the USEA-restored sensation as feedback in addition to proprioceptive feedback from intact muscles of the forearm and/or efference copy to determine hand position (see Additional file 3: Video 3, median trial time for video trials: 7 s, IQR 3.25 s, trial timing unavailable for other trials). Of the 6 failed trials, 2 resulted from timeouts and 4 resulted from misclassifications. The subject’s successful classification of ‘close’ versus ‘far’, along with subject’s verbal report of differences in the muscular effort required to move to the different positions, suggests that the subject could use residual function proprioceptive feedback or efference copy to identify his hand position (as distinct from the cutaneous sensory percept evoked by the USEA stimulation and used as a ‘stop’ signal), despite not being explicitly provided by the experimenters.Additional file 3: Video 3.Video3-Closed Loop Control S3. In this video, S3 used a cutaneous sensation on his ring fingertip (evoked by stimulation of a single ulnar-nerve USEA electrode) as feedback during an online, 1-DOF decode of 4-finger flexion/extension (decode via median-nerve USEA recording, driven by both neural and EMG). In the absence of visual feedback from the computer monitor, the subject successfully encountered and identified the location (‘close’ or ‘far’) of virtual targets in 41/47 trials (*p < 0.001,* binomial test), using the USEA-restored sensation as feedback in addition to proprioceptive feedback from intact muscles of the forearm and/or efference copy to determine hand position. (MP4 6230 kb)

**Table 3:** Confusion matrix for a 1-DOF closed-loop experiment performed by S3. ^a^ denotes that one “Far” trial resulted in a timeout which was scored as a misclassification. In this trial, the subject did not acquire the target and provide an answer in the allotted 30 s trial time

Response				
Condition	Close	Far	Totals	Accuracy
Close	9	1	10	0.90
Far	2 ^a^	13	15	0.87
Overall:			22/25	0.88

### Subjects describe their experience in a positive manner

Both subjects appeared to enjoy the experiments, as evidenced by their verbal expressions and eagerness to volunteer again for future studies. When asked if the USEA stimulation was something he would want to continue simply because it felt good, S3 responded: “Yeah. I would like it if you could keep it stimulated.” Following an online decode, S4, whose hands had been amputated 16 years prior, stated, “[…] when I tried to move my thumb and the thumb moved on the screen—that was the coolest thing that’s happened to me in 16 years.”

### Limited adverse effects

S4 developed an implant-related infection 4–5 weeks post-implant, from which he fully recovered, and from which he suffered no long-term deficits. Both subjects reported no long-term functional deficits due to the procedure, with a full return of phantom hand function to its pre-implant state after explantation of USEAs (data not shown).

## Discussion

We used USEAs implanted in peripheral arm nerves to: 1) provide subjects with independent, proportional position control of movement of many degrees of freedom via a virtual prosthetic hand (5 DOFs in formal testing, up to 12 informally); and 2) evoke numerous meaningful proprioceptive and cutaneous percepts across subjects’ phantom hands (up to 131). The number of DOFs achieved and the number of percepts evoked are greater than achieved in previous work with USEAs or other neural interfaces after amputation. We also provided one subject with limited closed-loop control of a virtual prosthetic hand. No long-term deficits were reported by the subjects after explant, although one subject experienced an implant-related local infection from which he recovered fully. Future implants should employ use of improved percutaneous site maintenance and/or wireless, non-percutaneous implants to help prevent infections. Both subjects appeared to enjoy having control over finger movements and experiencing phantom hand sensations evoked by microstimulation.

### Impedance

These results suggest that some USEAs may maintain a low-impedance condition in future long-duration implant studies, potentially allowing for chronic use of multi-channel neuronal recordings for decoding movements and intraneural stimulation for providing sensory feedback. However, failures potentially may occur at the electrode level, the wire-bundle level, or the connector level. Failure rates may be improved in future implants with improved external connectors, additional strain relief for USEA lead wires, and wireless devices.

### Decode

Both subjects demonstrated proportional control of a virtual prosthetic hand via multi-DOF decodes. S4 had higher-DOF decodes compared with S3. This improvement may have been due, in part, to implanting the USEAs proximal to the motor nerve branches to extrinsic hand muscles. Additionally, the higher amplitude EMG signals recorded from the distal site of S3 masked much of the neural activity, limiting the performance of online and offline decodes for S3. Similar EMG spiking has previously been reported for intraneural recordings [4]. Further offline processing revealed that neural activity can be recovered from the USEA signals using virtual referencing techniques [35].

Online decodes were driven by neuronal activity in S4, which, when decoded with a Kalman filter, provided independent proportional control of numerous movements (5 or more DOFs). This is in contrast to past approaches using EMG signals and/or classifier decodes, which have been limited to only 4 DOFs [36]. Furthermore, neural decodes offered control of several intrinsic hand and thumb movements, which would be inaccessible using EMG recordings from a typical amputated arm. In contrast to past, low-channel-count neural interface decodes, such as those performed using LIFEs and TIMEs, which have been limited to 3 DOFs, the high channel-count of the Utah Electrode Array has allowed us to provide subjects with high-DOF decodes (12 DOFs, informally), allowing restoration of control of combination movements and dexterous finger manipulations.

We also demonstrated that combination movements can be generated using an online Kalman decode trained with a limited number of simplistic, single-DOF movements. Training was performed in less than 5 min. Kalman decoding of this training can provide subjects with meaningful control of complex hand grips, pinches, and grasps as well as control of individual DOFs. In chronic implants, the short duration of training is important because training sessions may need to be carried out on a daily or weekly basis. The ability to generate novel grasps on the basis of simple training sets increases the functional range of useful movements for activities of daily living without increasing the time necessary for training.

Future improvements to online decodes should include incorporation of automated electrode selection algorithms, which improved performance of offline decodes performed after explant. Successful decodes leveraged information from sub-populations of several USEA electrodes, in contrast to using single channels for each movement. However, we observed subjectively that inclusion of too many USEA channels generally seemed to result in poor decode performance. An effective automated electrode selection algorithm should select all channels that produce relevant activity, while excluding channels that have no activity or new information relevant to the movements. In subsequent work, we have begun to implement on-line automated channel selection [18, 19, 37].

The ability of the USEAs to record neural activity in S4 substantially declined from post implant day 13 to 30. The decline may have been due to the decreased number of working electrodes, foreign body response to the USEA, array movement relative to the nerve, and/or fluid buildup in the intraneural space resulting from infection. The periphery is a harsh environment for the USEA. The median and ulnar nerves are subject to stretch, torsion, and compression during elbow bending, which was not restricted for S4. Such perturbation may cause the array to shift or pull out of the nerve. Due to the proximal location of the implant in S4, histological analysis of the intact array and nerve segment could not be performed to investigate array position with respect to the nerve at the end of the study. Additionally, MRI could not be used to image the arrays in situ as the USEA is not MRI-compatible. Although we have demonstrated the feasibility of using neural recordings from a peripherally-implanted USEA for real-time multi-DOF decoding, the general inability to detect motor-driven neural activity 30 days after implant precludes the use of USEAs for recording purposes in a commercially-viable prosthetic hand.

### Stimulation

Microstimulation via USEAs produced a rich selection of up to 131 different proprioceptive and cutaneous percepts spanning the hand. USEA stimulation required no long-term training or re-association or substitution of sensations. Proprioceptive percepts included flexions and extensions of each finger, flexion of the thumb, several intrinsic finger movements, and wrist extension. The improved ability to produce proprioceptive percepts in S4 compared with past subjects was likely due to placement of USEAs proximal to extrinsic hand muscle motor branches in S4.

In addition to restoring much of the functionality of an intact hand to amputees, quasi-continuous restoration of the sense of proprioception and cutaneous touch may help amputees perceive their prosthesis as an embodied replacement limb rather than a tool [1], which may decrease prosthesis rejection rates and improve amputees’ perception of the usability of the device [38]. Our subjects seemed to appreciate both the cutaneous and proprioceptive sensations evoked by USEA stimulation.

The high percentages of percepts in expected median and ulnar distributions suggests that cortical boundaries between median- and ulnar-nerve innervation regions for these subjects were still partially intact despite the amputation greater than 16 years prior. However, some projected fields for USEA-evoked cutaneous percepts spanned the edges of two adjacent digits, suggesting the possibility of blurring of digit boundaries in cortex. Importantly, proprioceptive percepts were more common in S4 compared with previous subjects, presumably due to implantation of USEAs midway along the upper arm, proximal to many nerve branches to the extrinsic hand-muscles.

We did not perform exhaustive testing of the effect of stimulation frequency on percept quality, location, intensity, and/or size. Future work should be performed to encode percept properties such as pressure gradations, joint angles, or joint velocities, via modulation of stimulation parameters, such as stimulation frequency. Additionally, activation of sub-populations of afferents with stimulation patterns faithful to each respective receptor type (e.g., slowly-adapting I type or II, rapidly-adapting type I or II, or group Ia or II intrafusal muscle fibers) may improve the naturalism, discriminability, and stability of percepts [39]. Naturalistic touch, such as the sensation experienced during motor task phase transitions, activates a diverse subpopulation of axons in distinct patterns, producing a fused population and temporal code [40]. In contrast to cuff electrodes, USEAs offer the opportunity to activate subpopulations of single axons in biofidelic patterns via independent control of stimulation via different electrodes, potentially offering unprecedented naturalism and variety in the nature of evoked percepts.

Notably, the stimulation thresholds for this study were mapped using the ascending method of limits. This method was selected to reduce the amount of time required to map the sensory perception threshold for the nearly 200 USEA electrodes, and because we were unable to reliably anticipate the expected level of the threshold values via USEAs prior to these subjects and wanted to avoid delivering overly strong, potentially painful stimuli. One disadvantage of this approach is that some observers may become accustomed to indicating that they do not perceive a sensation during initial subthreshold stimuli, which may result in a higher false-negative reporting rate when peri-threshold amplitudes are reached. For other observers, the opposite phenomenon may occur, in which the observer may make a pre-mature judgement of arrival at threshold (increased false-positive reporting rate at peri-threshold amplitudes [41]). Alternative approaches that at least partially resolve some of these limitations include the staircase procedures or averaging of the thresholds identified with the method of ascending limits and the method of descending limits.

Instabilities of percepts over time may be due to movement of the USEA electrodes relative to nerve fibers or due to the tissue foreign body response. Both potential issues may be ameliorated as improvements are made to the implantation procedure and the USEA materials and structure, and with longer implant times as processes reach asymptote. Further research is warranted to investigated and potentially improve USEA stability over time. Improved sensory percept stability for USEA-evoked sensations will need to be demonstrated for them to be functionally useful as a source of prosthesis sensory feedback.

### Closed-loop control

This is the first use of USEAs for closed-loop control of a virtual prosthetic hand in transradial amputees. Future closed-loop control with multi-DOF decodes and several unique sensory percepts may allow for dexterous manipulations with a prosthetic hand. Although we did not provide USEA-evoked proprioceptive feedback during closed-loop control for these subjects, we anticipate that this capability may be important in cases where the prosthesis encounters external counterforces, or when velocity control is desired (instead of position control). Importantly, improved methods for evaluating the extent and usefulness of closed-loop prosthesis control, including comparisons with control trials, need to be developed and implemented.

Ultimately, we foresee development of a portable, wireless system (i.e., no percutaneous wires) with USEA-enabled closed-loop control of a physical robotic hand that subjects may take home for use in activities of daily living [42]. Closed-loop control of multiple DOFs of a robotic prosthetic hand with graded feedback from multiple cutaneous and proprioceptive sensors via USEAs may allow users to perform activities of daily living while paying little visual attention to their prosthesis, or engage in tasks for which visual feedback is not readily possible (e.g., grasping the back side of an opaque object). In addition to restoring lost function, chronic use of such a device may transform subjects’ perception of their prosthesis from simply being a useful tool to being an integral part of their body. Embodiment of a prosthesis may not only reduce prosthesis rejection rates, but may also alleviate phantom limb pain and contribute to a restored sense of well-being and completeness [43–45].

The subject’s ability to correctly classify ‘close’ versus ‘far’ degrees of hand closure in the present study implies that he could successfully use of proprioceptive feedback from residual extrinsic hand muscles in the forearm, without these signals having been explicitly provided by the USEA neural interface. USEA-evoked stimulation in this case may have simply been used by the subject to know when to stop moving the virtual digits, and the assessment of whether the target was ‘close’ or ‘far’ could have been made based on the timing until perception of the USEA stimulation. Importantly, proprioceptive feedback would not be present for intrinsic hand muscles, or in the case of transhumural amputation (for which extrinsic hand muscles would be missing), or in the case where external forces deflected the position of the prosthetic hand, suggesting that neural interfaces that provide proprioceptive feedback could still prove useful. Future assessments of the usefulness of closed-loop sensory feedback should include USEA-evoked proprioceptive feedback as well as control trials in which the task is attempted without USEA-evoked sensory feedback.

### Study limitations

There are several limitations to this study including a small sample of two subjects, short implant durations (< 6 weeks), confounding factors to the online decode performance, the unstable recording/stimulating ability of the USEAs, and limitation of conclusions regarding the utility of USEA-evoked feedback during closed-loop control. Short implant durations do not allow for the long-term assessment of electrode recording or stimulation stability. Confounding factors to online decode performance include the number of available units on a given day, the ability of the experimenters to visually identify correlated firing rates, and on the ability of the subject to accurately repeat movements during training sessions, which may change over time. Such limitations are partially addressed in subsequent and ongoing, longer-duration studies [18, 20, 46]. Results of the present study are intended partly as proof-of-concepts, rather than demonstrating long-term viability or full functionality in activities of daily living.

## Conclusions

We have demonstrated that recording and stimulation via multiple USEAs implanted in the peripheral arm nerves 3 of human amputees can provide subjects with both 1) simultaneous proportional movement control of the digits and wrist of a virtual prosthesis; and 2) a rich selection of proprioceptive and cutaneous sensations spanning the phantom hand. Our achievement of a 5-DOF decode and 131 USEA-evoked cutaneous and proprioceptive percepts exceeds what has previously been accomplished with neural implants in the peripheral nerves of transradial amputees. Furthermore, we demonstrated that USEA stimulation and recording can be used for closed-loop control of a virtual prosthesis. Further investigation is warranted to demonstrate meaningful and repeatable closed-loop prosthesis control. No long-term functional deficits were reported by our subjects, although the implant did lead to a local infection in S4 that resolved with antibiotic treatment and explant of the devices. The subjects described the microstimulation-evoked sensations on their phantom hand and moving the virtual prosthesis in a positive manner. However, improved stability of these sensory percepts will be necessary in order for them to be functionally useful for prosthesis feedback. Future work will include development of automated channel selection and improved signal pre-processing algorithms for movement decodes, and use of biofidelic stimulation patterns and encoding of percept intensity gradations for sensory encodes. Ultimately, we expect that USEA-restored sensation and motor control could be used in closed-loop as part of a robotic upper-limb prosthesis that amputees may take home for use in activities of daily living.

## Abbreviations

DOF: Degree of freedom
EMG: Electromyogram
IQR: Interquartile range
RMS: Root mean square
S1-S4: Subject 1 – Subject 4
USEA: Utah Slanted Electrode Array
